# Experience of moral distress among doctors at emergency departments in Stockholm during the Covid-19 pandemic: a qualitative interview study

**DOI:** 10.1080/17482631.2023.2300151

**Published:** 2024-01-23

**Authors:** Clara Brune, Janne Agerholm, Bo Burström, Ann Liljas

**Affiliations:** Department of Global Public Health, Karolinska Institutet, Stockholm, Sweden

**Keywords:** Moral distress, moral stress, emergency departments, hospitals, Covid-19

## Abstract

**Purpose:**

The COVID-19 pandemic and consequent strain on healthcare globally shed light on the concept of moral distress among healthcare workers, albeit to a smaller extent among doctors at emergency departments. This study aimed to examine moral distress as perceived by medical doctors working at emergency departments in Stockholm during the pandemic, with the purpose of investigating causes of moral distress and methods to manage moral distress.

**Methods:**

Semi-structured interviews were conducted with twelve doctors working at two emergency departments. A questionnaire was developed based on previous research and the interviews were analysed qualitatively through thematic analysis.

**Results:**

The themes ”The factors that precipitated moral distress”, “Experience of workplace support” and “Coping strategies” as well as seven subthemes and 15 codes were identified. The informants reported on various situations with different causes of moral distress. Common causes were resource depletion, such as hospital bed shortages, and following stricter triage criteria. Informants reported varying ways of managing moral distress.

**Conclusions:**

Informants experienced moral distress when faced with challenges such as resource depletion, rules and regulations, and colleagues’ decisions. The informants who chose to seek support received it from their workplace, which helped them cope with their experiences. Some informants chose to not seek support.

## Introduction

Globally, the COVID-19 pandemic has forced healthcare systems to manage a dramatic increase in patient visits and hopital admissions. Healthcare workers have rapidly had to adjust to a new disease and develop best practice in how to handle new and complex care situations (British Medical Association). Further, infected, and absent staff has led to more strenuous working conditions and heavier workload. This has created strained situations in many healthcare settings, which increases the risks of moral distress (British Medical Association; Gustavsson et al., 2022). Moral distress refers to the feelings of unease that arises in situations when someone knows the morally correct action to take but is constrained from taking this action (Jameton, 1984). Repeated exposure to such situations increases the likelihood of experiencing moral distress (British Medical Association; Gustavsson et al., 2020). Moral distress is associated with job burnout (Epstein et al., 2020), depression, decreased job satisfaction and compassion fatigue as well as mental illness (Canadian Medical Association). It does not only affect healthcare staff but also patients, as moral distress may cause errors in patient care (Canadian Medical Association). Thus, examining, assessing, and addressing moral distress is essential both for healthcare staff and patients (British Medical Association).

There is a growing body of evidence showing that the COVID-19 pandemic has caused an increase in moral distress among healthcare workers (British Medical Association; Canadian Medical Association; Lamiani et al., 2021; Spilg et al., 2022). Identified risk factors for moral distress include lack of resources, control, and adequate preparedness (Gustavsson et al., 2020), issues commonly reported during the COVID-19 pandemic. For instance, a UK-wide survey showed that 97% of doctors who met only patients with COVID-19 reported experiencing moral distress, compared to 84% of those who met non-infected patients, and 88% of those who met both non-infected and Covid-infected patients (British Medical Association). The survey also reported that 96% of the doctors experienced that moral distress had increased during the pandemic (British Medical Association). A survey conducted in Sweden during the pandemic by Gustavsson et al. including all healthcare occupations, found that 85% of those who had worked with direct COVID-19 care experienced moral distress (Gustavsson et al., 2023), supporting the findings of high levels of moral distress in the British survey. In the British context, emergency doctors reported having experienced moral distress prior to the pandemic to a greater extent (81%) than other care specialities (60%) (British Medical Association). The main causes of moral distress in emergency departments (ED) both before and during the pandemic were reported in the survey to be insufficient staff, mental fatigue, inability to provide timely treatment, lack of time to give sufficient emotional support to patients, workplace culture and lack of personal protective equipment (PPE). Even though the same causes of moral distress were reported before, they were considered to be a larger problem during the pandemic (British Medical Association). Further, another study found that moral distress was generated in EDs as pandemic stressors threatened the sense of being a good doctor. The stressors were exemplified as limited healthcare resources, intensified patient triage, changeable selection criteria, limited therapeutic/clinical knowledge, and patient isolation (Lamiani et al., 2021). Another study reported that well-being has decreased while symptoms of stress have increased among healthcare workers in emergency departments during and after the first wave of the pandemic (Hesselink et al., 2021). Examining moral distress to identify triggering factors of illness is important to maintain the key role that the ED has in the care chain, as the health and well-being of the healthcare professionals is essential to have a fully functioning care system. Addressing moral distress in physicians is fundamental to ensure they can provide the healthcare needed, particularly during extraordinary circumstances. Additionally, increasing the well-being of healthcare workers is an important public health aspect in tackling the COVID-19 pandemic, and in general (Lai et al., 2020). Whilst moral distress has been reported to be present in emergency departments (Lamiani et al., 2021), to date, no studies on moral distress have been conducted in Swedish emergency departments. The survey conducted by Gustavsson et al highlighted that moral distress indeed was a burden among Swedish healthcare workers during the COVID-19 pandemic. However, no published previous studies in Sweden have focused on emergency medicine physicians or been conducted with in-depth interviews to outline the mechanisms behind moral distress in the Swedish context. Therefore, the aim of this study was to examine medical doctors’ perceptions of moral distress related to their work in the ED during the COVID-19 pandemic, with the purpose of investigating causes of moral distress and how physicians handled the moral distress they experienced.

## Methods

### Study design and setting

The study was an explorative qualitative study based on semi-structured interviews with medical doctors at two hospital emergency departments, purposively sampled with one hospital in central Stockholm and one outside central Stockholm, the capital of Sweden. Stockholm was chosen as it was particularly hard hit by the pandemic in Sweden considering mortality and morbidity in COVID-19 compared to other regions in Sweden (Socialstyrelsen).

### Participants

Two managers at the hospitals’ EDs identified through the researchers’ network were provided with information about the study and approved in writing that the interviews of their employees could be conducted. The managers further helped spread information about the study to doctors working at the EDs. Potential participants were identified and information about the study, the concept of moral distress and their confidentiality in participation was sent out via email. Those interested in participating were asked to contact project leader CB via email or telephone, meaning that the study sample was based on interest from participants. Those who had expressed interest in participating were then telephoned to arrange for an interview at a date and time convenient to them. They were also given the opportunity to ask questions about the study and their participation. Ethical clearance was not required under Swedish law on ethical review (Lag 2003:460 2§) as the study was undertaken as a student’s degree project. The participants gave formal written consent through Appendix A, or audio-recorded oral consent to participate in the study before the interview started and confirmed that they had received information regarding their right to drop out of the study at any point without having to provide an explanation why (Appendix B). This was emphasized so that participants felt comfortable dropping out if they were in any way negatively affected by the interview. The participants were informed at the start of the interviews that they did not have to answer all questions and that if they felt uncomfortable in any way, the interview would be paused or cancelled at any point. Interviews were conducted and performed until saturation was reached, as no more codes were identified in the analysis of the last interview.

### Interview guide

An interview guide (Appendix E) was developed based on questions used in previous studies on moral distress, and topics identified in existing literature. Following a few general questions on moral distress, the interview guide focused on the participant’s experience of moral distress in the emergency department. A pilot interview was conducted, after which two questions exchanged order to enhance flow and logic in the questionnare. Questions were asked about the pandemic in general and did not specify any time period of the pandemic. Two interviews were analysed before conducting the rest of the interviews, however, the interview guide was not changed after the analysis.

### Procedure of the interviews

The interviews were conducted by CB (female), a medical student with basic training and experience in interview techniques. Some participants had met CB during her clinical placement at one of the hospitals. Twelve medical doctors (8 residents, 3 specialists and 1 licenced doctor) expressed an interest in participating and all of them were interviewed with no dropouts. The interviews (6 interviews at each ED, one per person) were undertaken between 15 December 2021 to 31 January 2022. Three interviews were conducted on Zoom due to COVID-19 restrictions or other practicalities. The remaining nine interviews were conducted at the participants respective hospital. Interviews lasted between 42 to 95 minutes (mean length 58 minutes) and were digitally audio-recorded and transcribed verbatim by CB. Identifiable information in the transcripts was de-identified. Field notes were taken immediately after each interview. It was ensured during each interview that participants had support accessible from their workplace if they were to experience any distress. Participants further received contact information to the researchers and were encouraged to contact them with any questions or concerns. Transcripts and preliminary results were shared with participants who were invited to give feedback and comments. No participants wished to change their transcripts or comment on the results.

### Data analysis

The interview data were analysed using thematic analysis as outlined by Braun & Clarke (Braun et al., 2006), with an inductive approach. The transcripts of the first two interviews were individually read and re-read, discussed, and analysed by two researchers (CB and AL). AL has a background in public health and extensive experience in qualitative research. As CB worked clinically (medical student), the researchers’ own assumptions and interpretations based on experiences were discussed during the analysis, to ensure reflexivity as part of the process. Tentative codes were identified and entered MS Word to see if similar codes were identified by both researchers. The codes were refined, and more codes were identified as more data were collected. If there were disagreements regarding codes, it was discussed, and new phrases and categories were developed in collaboration. The tentative codes from all transcripts were further reviewed by both authors. Data sets were extracted and then combined into condensed meaning units, which were sorted into codes containing the core messages. The data set, organized by codes, was divided into preliminary themes and subthemes. The codes, subthemes and themes were further discussed by CB and AL and minor codes and subthemes were combined and applied to two interviews. The codes, subthemes and themes were further revised and agreed by CB and AL, and reviewed by a third researcher, JA, who has a background in public health and extensive experience in qualitative research. The remaining interviews were coded by CB and reviewed by AL. Examples of consistency between codes, subtheme and theme is reported in Table I. Consistency between the data presented and the findings are presented in Appendix C.

**Table I.** Identification of codes, subthemes and themes according to qualitative thematic analysis.

Meaning unit	Condensed meaning unit	Code	Subtheme	Theme
*” … he [the patient] would definitely qualify for intensive care, but it wasn´t possible—there were no hospital beds.” Participant 2*	The patient could not get the intensive care needed due to a lack of inpatient beds.	Shortage of inpatient beds	Situations caused by scarce resources	The factors that precipitated moral distress
*“ … there was a stress about how we would divide the patient flow to not spread infection, you don´t want people to get infected in the ED. It´s a stress related to the premises.”* *Participant 10*	Stress among staff regarding how to protect patients from infection in the ED.	Shortage of separate rooms to minimize spread of infection	Situations caused by scarce resources	The factors that precipitated moral distress

## Results

### Information about the participants

Relevant data was compiled into a table to present the participants position, years of experience and employee status during the pandemic (Table II). The number of years the participants had worked in the ED ranged from three to ten years. The participants had finished their internship or were residents or specialists. Four of the participants were female and eight were male.

**Table II.** Descriptive information about the participants.

Informants	Gender	Position
1	Male	Specialist
2	Male	Resident
3	Male	Resident
4	Female	Resident
5	Male	Resident
6	Male	Resident
7	Female	Licensed doctor
8	Male	Resident
9	Male	Resident
10	Male	Specialist
11	Female	Resident
12	Female	Specialist

### The participants’ definition of morality

**Figure 1.**
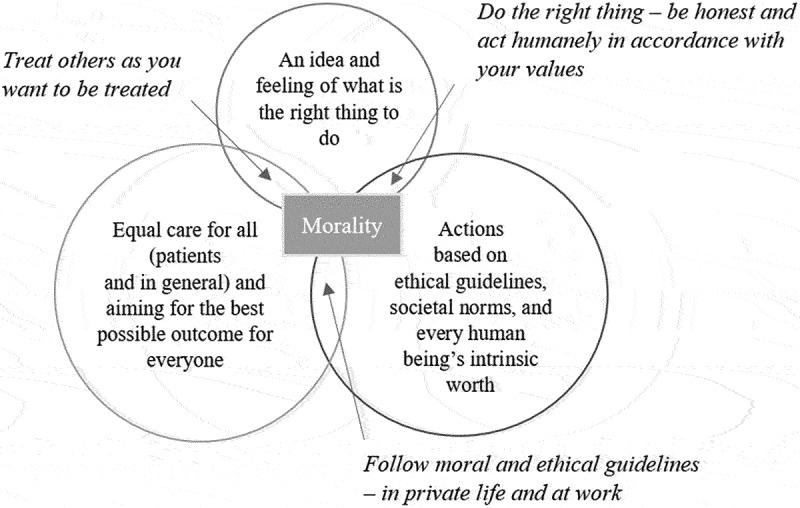
Illustration of the participants’ definitions of morality and their moral convictions.

At an early stage of the interview, the participants were asked to share their thoughts on what “morality” means to them (Figure 1). Recurring thoughts included to follow ethical guidelines in private and at work, treating everyone equally, following your inner guide regarding the right thing to do and treating others as you would like to be treated. The participants were then presented with the definition of moral distress (see Introduction), which all considered appropriate. If there had been disagreements regarding the definition, this would have led to a discussion with the participant and following mutual agreement on what definition to use during the interview.

### Situations perceived as morally distressing

The participants were asked to describe an incident they perceived as morally distressing at their work during the COVID-19 pandemic including how they had acted and more specifically what in the situation they experienced as distressing. If the participants had several incidents to describe, they were asked the same questions for each situation. The situations the participants reported on are summarized in Appendix D.

### Themes, subthemes, and codes

The thematic analysis generated three themes (in bold), seven subthemes (underscored) and fifteen codes (in italic) which are listed in Table III and presented below.

**Table III.** Themes, subthemes and codes.

Themes	Sub-themes	*Codes*
The factors that precipitated moral distress	Situations caused by scarce resources	*Shortage of inpatient beds*
		*Shortage of separate rooms to minimise spread of infection*
		*Heavy workload (not enough time or staff)*
		*Shortage of PPE*
	Rules and regulations conflicting with work ethics	*Following stricter triage criteria*
		*Rules about not visiting a dying relative*
		*Unfair prioritising of patients in conflict with the law*
	Decisions taken by colleagues	*Colleagues’ decisions causing challenging situations*
Experience of workplace support	Social support	*Receiving support from colleagues*
	Information/knowledge about the situation	*Lack of information and knowledge about the disease*
		*Changing guidelines*
Coping with moral distress	Coping strategies	*Accepting the situation and moving on*
		*No self-reproach*
		*Learning from negative experiences*
	Lack of coping strategies	*Compassion fatigue*

#### Theme 1: the factors that precipitated moral distress

##### Situations caused by scarce resources

###### Shortage of inpatient beds

All participants reported a shortage of inpatient beds as a problem that negatively affected their work, the patients, and the hospital. Some participants reported that the lack of beds caused moral distress.

If the decision is made based on hospital bed depletion, then there is a lack of material or staff or something else, which forces you to decide something that is not medically related—that´s what the moral distress is about I think. Participant 7

Some participants reported that moral distress arose in the decision-making of patient admission, when the admission criteria were not met but the doctor’s gut feeling told them otherwise, or when multiple patients needed a bed yet only one bed was available. The participants expressed that they wanted to keep beds unblocked and not burden colleagues, who they thought were already taking care of too many patients. Though the participants worked at the ED, several of them reported that the shortage of beds in the intensive care units (ICU) had been morally distressing. Some participants also reported on situations where patients had fallen severely ill due to depletion of hospital beds in the ICU.

Several participants further reported that a shortage of inpatient beds was a cause of moral distress since this meant that patients were kept in the ED while waiting for admission, even though the doctor had decided to admit the patient. This resulted in insufficient nursing since the ED is not supposed to nurse patients to a larger extent and for a longer time.

###### Shortage of separate rooms to minimise spread of infection

Several participants reported experiencing moral distress due to the risk of spreading infection between patients.

… it always felt horrible when you put a patient, because there were no rooms, in a corridor or a booth, and they would lay there and cough on someone who was there because of, for example, a hip fracture … Participant 2

###### Heavy workload (insufficient time or staff)

Several participants reported that a heavy workload in the form of many patients yet no time for all of them caused moral distress and generated other incidents where moral distress arose. For instance, several participants described situations where critically sick patients were left unattended for a longer period of time due to understaffing and work shifts with high levels of staff missing. Several participants, particularly in the hospital in central Stockholm, reported that patients had to wait longer than the guidelines on waiting times allow for certain conditions, before seeing a doctor.

… for me, much of the moral distress is when I meet someone at 10 PM who came in at 10 AM, for me it´s really tough to know that the person I´m meeting now has waited for 12 hours, and I might say ‘go home’ after 5 minutes … Participant 2

Moral distress was specifically reported related to situations when the emergency alarm had been triggered, meaning that a patient needed emergent care by a healthcare team which the doctor was part of.

And then you worry about other patients that you have to leave. Sometimes you receive a patient in the ER, stabilize them, and then the next alarm sounds. By then you don´t know whether the first patient has everything they need. Participant 3

###### Shortage of PPE

Most participants reported having experienced moral distress due to insufficient personal protective equipment (PPE), particularly at the beginning of the pandemic.

We hid it so that we would have masks for the weekend, in locked rooms so that it wouldn´t be used up unnecessarily. From time to time there definitely was a depletion. There were some who bought raincoats and their own masks. Participant 3

The participants explained that due to PPE depletion, the consultations were minimized to one visit, making the clinical judgement more complicated and insecure, which caused moral distress.

##### Rules and regulations conflicting with work ethics

###### Following stricter triage criteria

Several participants reported that stricter triage criteria across the hospital during the pandemic caused moral distress. Yet, some thought that clear guidelines facilitated decision-making and were protective against moral distress.

… we triaged pretty strictly at the start of the pandemic, and sent people home without a real clinical judgement, people who we otherwise would have taken into the ED and assessed … Participant 1

###### Rules about not visiting a dying relative

Some participants reported that having to follow guidelines such as the curfew, meaning that relatives could not visit to reduce the spread of infection, was a source of moral distress, specifically related to dying or sick patients and patients who needed support from their relatives.

[The patient] had had covid for a week. This was the first wave, and our experience was that basically everyone died. He was very sick, so I called his children and told them. They wanted to come and visit, but they simply weren´t allowed. I admitted him, and he died the day after. Participant 5

In these situations, some participants reported that they had broken the rules and not followed guidelines, and therefore prevented feelings of moral distress.

###### Unfair prioritising of patients in conflict with the law

Some participants reported that unequal care was a result of stricter triaging and that patients were admitted depending on the availability of hospital beds rather than the patients’ medical condition. The participants reported that this conflicted with ethical standards and the Swedish Healthcare Act, as healthcare was not provided on equal terms and based on need.

[in such situations] you should never, you´re not supposed to decide or discriminate based on age, but you started to … Participant 2

##### Decisions taken by colleagues

###### Colleagues’ decisions causing challenging situations

Disagreement on treatment limitations made by intensive care doctors, and not understanding why and how these decisions were made was reported to cause moral distress by several participants.

“However, we don´t know how they [ICU staff] judge, which means that when you get into ethical conflicts, it´s probably due to incomprehension. You don’t always know how the ICU resonates. They may come down and be in a hurry and judge that ‘this is not an ICU candidate’, without having discussed why it isn´t.” Participant 6

#### Theme 2. Support from the workplace

##### Social support

###### Receiving support from colleagues

Several participants had reached out to colleagues for support and received it. Some participants were burdened by their experiences and had not sought support, but reported that support was always accessible from colleagues and supervisors, and organized if needed.

##### Information/Knowledge about the situation

###### Lack of information and knowledge about the disease

Several participants reported working with insufficient information about Covid-19, specifically at the beginning of the pandemic. Not having enough information about how to act and what guidelines to follow caused of feelings of frustration and contributed to the worry of getting infected and infecting others.

####### Changing guidelines

Most participants at the ED in central Stockholm reported that changes to the routines and guidelines were frequently made by more senior staff, causing confusion and frustration. Contrary, several participants in the hospital outside central Stockholm reported being consulted about changes related to daily routines, guidelines and reorganization, which they reported as something positive.

It has been very messy overall … We´ve changed our workways and flows several times since then, you can barely keep up. Participant 6

… all decisions have been anchored with the clinicians and revised if something hasn´t worked and nothing has been run over our heads. Participant 11

#### Theme 3. Coping with moral distress

##### Coping strategies

###### Accepting the situation and moving on

Several participants reported talking to colleagues as a coping strategy that helped. Several others reported that they had moved on without support or had experienced negative feelings but pushed themselves to move on, because they felt that they had to. Several participants thought that following an incident they must move on due to ethically challenging situations being a part of their work, accepting moral distress and acclimatizing to the constant exposure.

You get used to it. You have gotten used to it, this is only one out of many … Informant 7

Some participants further expressed that they had to adjust their ethical standards to meet their workplace reality.

I think it´s a survival instinct, because I don´t think people who principally keep their full ethics survive here. Participant 5

Several informants reported that they moved on in the moment during their work shift by focusing on the next patient and that they did not have time to reflect on the morally distressing situation.

It was tough, but you keep working. Informant 12

Some informants expressed that they had not dealt with the morally distressing incident, or that they had avoided it.

“I haven´t coped with it.” “I should have thought about it. My strategy has rather been to forget and repress it.” Informant 3

[I handled it] Not at all, I pushed it away. Informant 5

###### No self-reproach

Some participants described how they had overcome incidents by not blaming themselves but accepting the limits of their responsibility when decisions were made by others.

… that it´s not something I´ve decided, but someone else’s forced decision. When it´s decided at a more senior level you can also refer to their decision—I´m not the one deciding this and am incompetent and abandoned my medical principles, this is forced upon me. Participant 12

Some informants referred to structural problems as a way to not blame themselves.

All work in the ED is about choosing, and I think the reason why I feel moral distress is that it doesn´t get as good as it should for the patients, but at the same time I find it pretty easy to think it´s not my fault. I think ‘this is a structural question, it´s an organizational question—I can´t be blamed for there being so many unattended patients in the ED or there being so many sick patients—I´m just me and I can only do what I can’, and then it gets easier to handle. Even though it´s not okay. Informant 11

###### Learning from negative experiences

Some participants reported constructive ways to learn from the turbulence at work during the pandemic, and reported that the only way to move on was to learn from each experience.

You have to try to carry them [the experiences] with you and learn from them. Become better, find better ways to do it next time. That´s the only way. Participant 4

##### Lack of coping strategies

###### Compassion fatigue

Several informants reported becoming cynical, as well as feeling bitter and experiencing compassion fatigue.

I´m thinking of the word compassion fatigue, that you consider situations that are very stressful as blasé because you can´t stand it every single time. Informant 3

… you become cynical quickly, it is obvious. It happens to everyone, I think. Informant 5

Some informants also reported that their moral compass had become blunt by the situations they experienced at work.

… in the end, you become pretty blind, and much of your moral compass becomes blunted at work … Informant 2

The cynicism that some expressed was directed towards the healthcare system not functioning properly, and reported that the problems stemming from it were the ones causing moral distress.

I think the hospital situation and the whole healthcare situation is stabbing against your moral. Informant 5

## Discussion

The study results show that doctors in the included emergency departments experienced moral distress during the COVID-19 pandemic. The participants reported experiencing moral distress in a wide range of situations. These were often caused by resource depletion such as hospital bed shortage, insufficient staffing, and lack of personal protective equipment. Participants were burdened by a heavy workload and subsequently experienced moral distress when having little flexibility in undertaking their job, and at the same time adhere to rigorous COVID-19 infection control and stricter triage criteria. Most participants experienced compassion fatigue and considered support from colleagues and various coping strategies as essential for moving on. Participants further reported that patients had been subjected to unequal care during the pandemic, as a consequence of resource depletion.

The inability to provide timely and optimal care combined with a high workload—which was mentioned by many participants—have been found in a recent study to be most generally distressing for healthcare staff internationally in EDs (Greenslade et al., 2020). Similarly, insufficient staffing was reported in another previously conducted UK-wide survey to be the most common cause of moral distress (British Medical Association). The findings support previous studies from several other countries (Lamiani et al., 2021; Rao et al., 2021) suggesting an international similarity in the difficulties healthcare workers in EDs have faced during the pandemic. Many aspects of moral distress reported were however not pandemic-specific. Thus, it can be speculated that the pandemic may not have created new areas of moral distress but highlighted existing causes of moral distress. This is supported by what several participants reported—that moral distress existed before the pandemic, but that it was highlighted by the strain and changed working conditions during the pandemic. This supports a British study showing that 60% of participating doctors had experienced moral distress at work prior to the pandemic (British Medical Association). All participants in the current study considered bed shortage as morally distressing, including lack of hospital beds in the ICU as it forced them to apply stricter triage criteria.

Being responsible for too many patients was also reported by several participants as both a common problem in the ED, and a cause of moral distress. When asked what could be done to reduce the amount of moral distress, the participants answered that more hospital beds, more resources, reduced workload with fewer patients, and more time would make a significant difference. The doctors’ experiences correspond with previous research (British Medical Association). It is possible that policy directed at mobilizing more resources and hospital beds could have a positive impact on the moral distress experienced by doctors in emergency departments. Some participants, all working in the hospital outside central Stockholm, reported having positive experiences from the pandemic because they could decide over their own working environment, and that the decisions being made were less bureaucratic. Such aspects have also been mentioned in a study on moral distress among doctors in the UK during the pandemic (British Medical Association), who further ranked “streamlined bureaucracy” to be the second most important intervention to reduce moral distress, after more staff. A possible implication may be to involve healthcare workers in the decision-making in their workplace, since this seemed to affect their view on work when experiencing job strain.

Apart from reporting insufficient care and nursing of the patients, a known cause of moral distress (Canadian Medical Association), participants also reported that repeated exposure to morally challenging situations could have a compounded negative effect. This correponds to findings from previous studies (Gustavsson et al., 2022). Repeated exposure may cause what is termed moral residue, a heightening of the individual’s level of moral distress in subsequent experiences (Canadian Medical Association). Whilst doctors can learn from medically challenging situations and develop in their professional role from them, morally challenging situations do not become easier to tackle with time and experience, and are harder to handle since there is no real ´solution´ in the original sense of the word (Larsson et al., 2007). Therefore, workplace guidance on morally distressing situations are important to enable healthcare staff to cope with moral distress, which has already been suggested for disaster responders to alleviate moral distress (Schwartz et al., 2012). Previous interventions to mitigate moral distress and burnout among clinicians have focused on the individual clinician (Rushton, 2013; Rushton et al., 2006, 2009, 2013). However, research has shown that leaders and organizations also have important roles in addressing the practical effects of moral distress, as mitigation needs to be initiated from an organizational level (Hertelendy et al., 2022) and as organizational support has been found to have effects on reducing moral distress (Rathert et al., 2016). Thus, institutional involvement is important both for mitigating the root causes of moral distress, as well as for providing support to healthcare workers who are exposed to it. In accordance with previous research, the authors of this paper underline the importance of developing functioning tools to manage moral distress.

The subtheme “Coping strategies” included the code “No self-reproach”. This code was partly generated by participants stating that they managed to not feel guilty over the situation through referring to their own limited area of responsibility. On the contrary, some participants reported that they had repressed their experiences and not handled them. They also reported that they had not sought support. The different ways of managing moral distress demonstrates that all individual clinicians may have different initial reactions to morally distressing situations. Although individual differences may signify different types of support needed and different short- and long term reactions to difficult experiences, moral distress and potential mitigating strategies need to be discussed on a structural level. All participants mentioned that they could receive support from their workplace if they reached out for it, and several participants had done so. As workplace support has been found to have effects on reducing moral distress (Gustavsson et al., 2020, 2022; Rathert et al., 2016; Spilg et al., 2022), access to support may potentially have had a protective effect on the participants. This could form the basis for a potential future intervention that aims to mitigate moral distress, a knowledge gap in research on prevention of moral distress and efficient coping mechanisms (Morley et al., 2021).

A major strength of this study is that it is a novel study in the Swedish context, and that it provides data on healthcare workers way of managing moral distress and support received. This may provide valuable information for further research on ways to manage moral distress, which has been pointed out as necessary in previous studies on moral distress (Gustavsson et al., 2023). Another strength is that participants were given the opportunity to read the transcripts and provide feedback on the preliminary results, and quote(s) for each finding have been provided to ensure consistency between the data and the findings.

## Limitations

Limitations to the study include that data were only collected at two sites, making it difficult to generalize the results. Yet, data were collected until data saturation was reached. Another limitation is the subjectivity of the researchers. The research team consisting of both clinicians and researchers in public health can however be considered a strength, as it added nuance to the analysis process and reflexivity as the researchers had to reflect on how their background influenced their interpretations of the results. Still, it cannot be guaranteed that the researchers’ perceptions and background did not affect the analysis, which is why sharing the transcripts and preliminary results with the participants for their comments, known as member checking, increases the credibility of the study (Birt et al., 2016). The examples of morally distressing situations given by participants included situations where the participants could not follow what they had defined as the morally correct action (Figure 1), supporting the finding that participants were in fact burdened by moral distress.

## Conclusions

In conclusion, the findings suggest that the interviewed doctors at the two EDs in Stockholm experienced moral distress during the COVID-19 pandemic when unable to provide equal and sufficient care for patients due to barriers such as resource depletion, rules and regulations, and colleagues’ decisions. Subsequently, the participants faced hardships and the patients were subjected to unequal care. Supportive colleagues were important for coping with the moral distress experienced. The results also suggest that involving healthcare workers in decision-making affects the way they view their work environment when experiencing job strain.

## Article summary

- **Why is this topic important?** This is the first qualitative study in Sweden examining the experience of moral distress among medical doctors working in emergency departments during the COVID-19 pandemic.
- **What does this study attempt to show?** Moral distress has been present and affected doctors working in emergency departments during Covid-19.
- **What are the key findings?** The participants experienced moral distress when unable to provide equal and sufficient care. Participants further experienced barriers to providing sufficient care due to the pandemic.
- **How is patient care impacted?** Participants perceived that patients were subjected to unequal care, which created moral distress among the study participants.

## Data Availability

Anonymized interview transcripts in the original language (Swedish) can be provided upon reasonable request by contacting the corresponding author.

## Geolocation information

Stockholm, Sweden

## Appendix A

### Consent form

Consent to participation

I have been informed orally and in writing and given the opportunity to ask questions about the study on moral distress among doctors in emergency departments in the Stockholm region during the COVID-19 pandemic.

I have also received information that participation is voluntary and that I can cancel my participation at any time. If I choose not to participate or wish to cancel my participation, I do not have to state why, without any subsequent consequences.

My answers will be processed in a way that unauthorized persons cannot access them. xxx is responsible for handling personal data such as names and contact details.

I hereby consent to the processing of personal data in accordance with the GDPR and to participate in the study.

─────────────────────────────────────────

Signature

─────────────────────────────────────────

Name              Contact information

─────────────────────────────────────────

Date

## Appendix B. Information sheet

Information sheet

### Research study on moral distress among doctors at emergency departments in Stockholm Region during the COVID-19 pandemic

Moral distress is the stress and following feelings that arise when you ethically and morally know how you should act, but are not able to. It can arise due to lack of resources or other barriers. Moral distress being present among healthcare workers was known before the pandemic, and there is research among intensive care workers during the pandemic. However, we know less about moral distress during the pandemic in other departments such as the emergency department. You are asked to participate in this study as I am writing my thesis as part of my medical studies about how the COVID-19 pandemic might have created and affected moral distress among doctors at emergency departments.

The study has a special focus on what connections there are between moral distress and the pandemic. I want to interview you if you choose to participate. The interview is estimated to take one hour and includes several questions which you choose if you want to answer. All identifiable information will be deidentified and your participation will be anonymous. University is responsible for the handling of your personal data according to GDPR. The study results will be analysed and presented in my thesis. We want to emphasize the following: It is completely voluntary to participate. You can at any point terminate your participation without informing why. The collected data will be handled in a way that no person can be identified. You have the right to take part of the collected information in the study, and have the right to get it changed if it is incorrect information about you. As part of the interview I will ask for your consent to participate. If you have any questions about the study, we ask you to contact us.

## Appendix C. Data corresponding to each code

**Table:**

Shortage of inpatient beds	”It´s challenging and morally distressing not just being able to follow guidelines and what you´ve learnt, but putting into the equation how many free beds there are. The treatment and admission should only be based on medical indication, which is why it´s morally distressing when you adjust your judgement after the current situation with inpatient beds.” Informant 9
Shortage of separate rooms to minimize spread of infection	“ … it always felt horrible when you put a patient, because there were no rooms, in a corridor or a booth, and they would lay there and cough on someone who was there because of, for example, a hip fracture … ” Informant 2
Heavy workload (not enough time or staff)	”It was one evening I think, when one nurse had to take care of three units by herself—and normally there is at least one nurse per unit, as minimum staffing in the summer, which itself isn´t enough.” Informant 3 “And then you worry about other patients that you have to leave. Sometimes you receive a patient in the ED, stabilize them, and then the next alarm sounds. By then you don´t know whether the first patient has everything they need.” Informant 3
Shortage of PPE	“We hid it so that we would have masks for the weekend, in locked rooms so that it wouldn´t be used up unnecessarily. From time to time there definitely was a depletion. There were some who bought raincoats and their own masks.” Informant 3
Following stricter triage criteria	” … we triaged pretty strictly at the start of the pandemic, and sent people home without a real clinical judgement, people who we otherwise would have taken into the ED and assessed … ” Informant 1 “There are many situations, mainly when standing on the borderline of ICU—no ICU with patients, where the threshold to qualify for intensive care before the pandemic was lower than during the pandemic, and it´s still very strained I would say… For example, the several who otherwise would have been tubed in the ER and taken to the ICU, but who under the circumstances not qualified for that type of care, due to current diseases. The boundaries have become stricter, and the discussions have become longer in the ICU.” Informant 6
Rules about not visiting a dying relative	“[The patient] had covid since a week back. This was the first wave, and our experience was that basically everyone died. He was very sick, so I called his children and told them. They wanted to come and visit, but they simply weren´t allowed. I admitted him, and he died the day after.” Informant 5
Unfair prioritizing of patients in conflict with the law	” … you should never, you´re not supposed to decide or discriminate based on age, but you started to … ” Informant 2 “I think the worst stress is that you maybe don´t give equal care to all patients. According to the healthcare law, the sickest should receive healthcare first and of the best quality and I think that it´s the ‘best quality’ that we often fail … I would say that the moral distress arises when you question the medical indications for admission, which are actually very clear.” Informant 9
Colleagues’ decisions causing challenging situations	“However, we don´t know how they [ICU staff] judge, which means that when you get into ethical conflicts, probably due to incomprehension. You don’t always know how the ICU resonates. They may come down and be in a hurry and judge that ‘this is not an ICU candidate’, but then it hasn´t been discussed why it isn´t.” Informant 6 “…that´s how it works, it´s their [ICU staff] department and their competence and they decide who to admit. Still you get angry *when things happen the way they do*.” Informant 2
Receiving support from colleagues	*“Yes, a new colleague came who started in the afternoon and who I trust, who got involved and made decisions that I felt were safe. Then I got the opportunity to talk it through with the colleague straight afterward and got support, so then it felt a lot better.” Informant 11*
Lack of information and knowledge about the disease	“I had no idea about how to act and what to do—I hadn´t gotten any information from the management because they barely knew themselves how we were supposed to handle this. Everything was so new and people were panicking, and then I felt that ‘okay, I´m supposed to be here to help and support patients who come in and want help, but I barely know myself how to act”. Informant 8 “ … in the beginning, there was a lot of worry. ‘When will I get infected? How will I get infected?’ because you saw people and heard about colleagues who got infected and even ended up in the ICU, and you were standing there in the frontline and were supposed to work with this unknown [virus].” Informant 9
Changing guidelines	”It has been very messy overall … We´ve changed our workways and flows several times since then, you can barely keep up.” Informant 6
Accepting the situation and moving on	“You get used to it. You´ve got used to it, this is only one out of many … ” Informant 7 “It was tough, but you keep working.” Informant 12
No self-reproach	“All work in the ED is about choosing, and I think the reason why I feel moral distress is that it doesn´t get as good as it should for the patients, but at the same time I find it pretty easy to think it´s not my fault. I think ‘this is a structural question, it´s an organizational question—I can´t be blamed for there being so many unattended patients in the ED or there being so many sick patients—I´m just me and I can only do what I can’, and then it gets easier to handle. Even though it´s not okay.” Informant 11
Try to learn from negative experiences	“You have to try to carry them [the experiences] with you and learn from them. Become better, find better ways to do it next time. That´s the only way.” Informant 4
Compassion fatigue	“I´m thinking of the word compassion fatigue, that you consider situations that are very stressful as blasé because you can´t stand it every single time.” Informant 3 ” … you become cynical quickly, it is obvious. It happens to everyone, I think.” Informant 5 “ … in the end, you become pretty blind, and much of your moral compass becomes blunted at work … ” Informant 2

## Appendix D. Morally distressing situations reported by the participants

**Table:**

Participant	Situation	Action taken	Experienced moral distress
**1**	Sending patients home without a proper clinical evaluation, and telling them that it is safe, but not believing it was safe.	Following guidelines even though the gut feeling says no.	The participant experienced that it felt unsafe and the clinical judgement felt incomplete and insecure, and that they lied to the patient.
**2**	Other staff in the ER treated an unvaccinated patient rudely.	Initially, the participant did nothing. Later, the participant tried to be kind to the patient and lead by example in treating the patient nicely.	The participant thought it was wrong of their colleagues.
**3**	The participant was the responsible doctor in the area with the most ill patients and got so stressed because the alarm phone kept ringing and because they had to attend to new alarms, that the participant left the most ill patients unattended for a long period of time.	The participant forgot to tell someone else to attend the area. The incident was not followed up.	Something bad could have happened and someone could have died. The participant felt that they should have followed up on the incident and written a deviation report.
**4**	A very ill patient in the ED needed palliative care the last hours living but could only receive it in the ICU, where there were no hospital beds. The cardiologist did not have time to come and help.	The participant tried to support their younger colleague who was responsible and tried to contact the ICU and cardiologist.	The participant described that it felt tough that the patient had to spend their last hours in life in misery and they drowned in their lungs because the clinicians could not help.
**5**	I) The curfew forced the participant to deny a patient’s family to come to the hospital and say farewell. II) Sending home sick patients because they needed the spare beds.	I) The participant followed the guidelines.	I) The participant experienced that letting patients die in the ED felt like stripping them of their human value. II) Sending patients home meant taking a risk on their behalf.
**6**	A patient with repeating seizures who needed intensive care had to wait for eight hours before they were admitted, which can result in long term brain injuries.	The participant tried to do the best they could. The participant was kept in the ER as there were no spots in the ICU. The doctors had to continue care for the patient in the ER even though they had many other ill patients who needed their attention.	The participant felt frustrated as this is usually a problem that can be solved quickly, but they could not because of a lack of resources.
**7**	A patient needed intensive care, but there were no available beds. Other patients were kept unattended while the participant had to give priority to alarms. The intensive care doctor didn’t have time to come and help.	The participant contacted other healthcare staff for help.	The participant felt like they did not have control over the situation.
**8**	I)There were specific guidelines on who should be tested, but the participant wanted to test and give full information to everyone. II) Restrictions due to logistic reasons and the law prevented the participant to give the patient the care they needed.	I)The participant did sometimes not follow the guidelines. II) The participant broke the rules and the law and risked their medical licence.	I)The participant sent people home without having helped them. The participant wanted to act in a different what than what they did. II) The participant had to choose between their safety as a doctor and the patients’ safety.
**9**	I)Strict criteria regarding who could be admitted. II) Triaging who could be let into the ED from a temporary tent outside. III) Ill patients who needed to be admitted to the ICU but remained in the ED because of a lack of beds.	I)Followed guidelines. II) Made a clinical assessment in the tent with limited tools. III) The participant had to attend to the patient and could therefore not attend to others who needed their help.	I)The participant did not follow what their gut feeling told them to do. II) The participants’ clinical judgement was not based on the fundaments learnt. They felt bad for the patients who were not met properly. III) Felt moral distress from not being able to help other sick patients.
**10**	Patients in the waiting room could infect others. The ED does not have premises that allow the clinicians to separate the patients.		The participant experienced loss of control.
**11**	I) Giving a patient bad news when they could not have family there to support them. II) An older colleague asked the participant to do something invasive on an old, sick patient.	I)The participant did not follow guidelines and let the family come anyway. II) The participant followed orders.	The participant experienced difficulty in I) Choosing between rules and what feels rightas they believe that people deserve to have close ones with them when receiving bad news. II) Determining was best for the patient. Afterwards, the participant felt that they could have acted differently.
**12**	Restrictions to avoid the spread of infection included for example avoiding inhalations. This meant that the patients did not receive the inhalations until they were admitted, which delayed the start of the treatment.	The participant followed the guidelines.	The participant noticed that the delay in receiving treatment affected the patient. The participant felt bad because they had a solution, but the guidelines said no.

## Appendix

1. Tell us very briefly what your work here at the emergency department usually entails.
2. How long have you worked here? What shifts do you work? (eg day/night)
3. How do you define morality?
4. How would you describe your moral values?
5. Are your moral values at work the same (as those you have as a private person)?
6. I will now read out a statement: My definition of morality is that which distinguishes between good and evil actions. The definition I use for moral distress is the negative feelings that arise when I cannot act in line with my ethical and moral values. In this case, ethical and moral distress are synonymous. What do you think about these definitions? Do you agree?
7. Do you normally experience a lot of moral distress in the emergency department?
  - Would you say that the amount of moral distress you experience in the emergency department has been affected by the pandemic?
8. Can you describe a situation during one of your work shifts since the start of COVID-19 when you felt that you could not follow and act based on your moral values? When was this? What happened?
9. Can you describe what in the situation was difficult and/or stressful?
10. What decision did you make in this situation?
  - What decisions did others make in this situation?
11. Did you have to make decisions outside of your area of expertise? If so: how has it affected you?
12. What do you think was the cause of the situation?
13. If you think back on the situation; how did it feel in the moment?
  - How did you handle this?
14. How did it feel afterwards?
15. Was there anything that made the situation easier?
16. Was there anything that made the situation difficult?
17. What kind of support did you receive from your workplace? When did you receive such support?
  - Were you able to talk to your colleagues, and did you get support from them?
  - Did you have any tools that supported your decision making?
18. How was your ability to work affected by being in such a situation/making such decisions?
19. Have you noticed any special strategies or behaviours in yourself at the time or afterwards as a consequence of the situation you experienced?
  - How do you feel when you think about the situation now?
20. When you come home or live your normal life as a private person, how does experiencing the difficult situation you described affect you?
21. Do you have more situations you want to tell me about? *If yes: Repeat from question 7*
22. Do you experience more moral distress if a patient does not speak Swedish as a native language?
23. Have you experienced that other language barriers/communication difficulties affect the experience of moral distress? If yes, how?
24. What could be done to reduce the risk of experiencing moral distress in emergency departments under both previous circumstances and during the pandemic?
25. How, if at all, were you affected in your work by the media’s reporting on Covid-19?
26. Did the intense media reporting affect your way of acting in situations when you experienced moral distress?
27. Has there been any reorganization or restructuring at your workplace as a result of the pandemic? If so, how have you experienced it?
28. Do you have anything to add?
